# Dr. Benjamin H. Catching

**Published:** 1900-01

**Authors:** 


### Obituary.

### DR. BENJAMIN H. CATCHING.

THE death of this prominent dentist was noticed briefly in the December number.

He died November 23, 1899, suddenly, of apoplexy, at his home, and while preparing to leave for his office.

Dr. Catching was born in Georgetown, Mississippi, in 1848, being fifty-one years of age at the time of his death. He moved to Atlanta, Georgia, in 1880, and resided there up to the period of his death.

His work in dentistry is, probably, better known than that of any member of the dental profession South. He was for a number of years editor of the *Southern Dental Journal*, and subsequently established "Catching's Compendium" and the *American Dental Weekly*.

The remarkable character of the man is best illustrated in the issue of the "Compendium." This, as its name indicates, was a condensed statement of dental periodical literature for the year. Few, perhaps, can realize what this meant in exhaustive labor. It probably had much to do in shortening his valuable life. Aside from the work entailed in its preparation, he must have had many hours of anxiety in regard to its success professionally and financially. A quotation from a letter to the writer of March 1, 1894, gives some idea of the laborious task he had set himself yearly to accomplish: "I send to-day a 'Compendium' for 1893. I hope you will like it. Now that the work on it is about over, I feel completely broken down. No one knows the immense amount of work it requires to get out the book."

At this period—1893—he adopted the suggestion of the writer to extend the scope of the book to cover the work of other nationalities. In the preface of this year's book he announced, "In this issue the condensed practical results of the dental journals of six different nations are given. The next edition will contain the practical results of the dental journals of the world."

The cause of the discontinuance of the "Compendium" is unknown to the writer, but possibly the lack of financial support combined with the exhausting character of the work may have compelled its abandonment.

The *American Dental Weekly* followed, but this lasted but a brief period. The day for weekly dental journals had not then arrived, and probably will not in many generations. It was well edited during the year it lasted, and was always a welcome visitant among our exchanges.

Thus at a comparatively early age one of the brightest men of the South has concluded his work. While his home was at Atlanta, his name was a household word throughout this country and well known abroad. While he loved his native South, he was broad enough to recognize that dentistry knows no clime and no people, but is part of the humanitarian work among all nationalities.

He was deeply interested during the later days of his life in the question of dentists in the army and navy, and probably one of the last, if not the last article from his pen was published in the November number of this journal upon that subject.

The writer enjoyed his friendship for many years, and, while personal association was infrequent, he always felt that he had received a portion of Dr. Catching's enthusiasm whenever that opportunity offered.

The South will miss him, the world will miss him, and the writer will continue to feel that in his death the silent reaper has ingathered one of the true-hearted, whose love for his profession led him to walk, all too early, up to the last and eternal sacrifice.

Dr. Catching leaves a wife and four children,—one son and three daughters.

